# Which Divergent Thinking Index Is More Associated With Problem Finding Ability? The Role of Flexibility and Task Nature

**DOI:** 10.3389/fpsyg.2021.671146

**Published:** 2021-07-22

**Authors:** Ahmed M. Abdulla Alabbasi, Roni Reiter-Palmon, Zainab M. Sultan, Alaa Eldin A. Ayoub

**Affiliations:** ^1^Department of Gifted Education, Arabian Gulf University, Manama, Bahrain; ^2^Department of Psychology, University of Nebraska Omaha, Omaha, NE, United States; ^3^Ministry of Education, Manama, Bahrain

**Keywords:** divergent thinking, ill-structured problems, creativity, task nature, problem finding, fluency, flexibility, originality

## Abstract

Problem finding (PF) and divergent thinking (DT) are considered to be indicators of creative potential. Previous studies, with different goals, suggest a positive correlation between PF and DT. However, none of these works have explicitly examined which index of DT is more associated with PF. The current investigation examined the association between PF and three main indexes of DT: fluency, flexibility, and originality. It also tested whether such a relation differs based on task nature (verbal vs. figural). The sample consisted of 90 sixth graders who completed three tests: (a) a verbal DT test, (b) a figural DT test, and (c) a PF test. Correlational analysis showed that flexibility was highly correlated with PF in the verbal DT test, whereas originality was significantly correlated with PF in the figural test. Results of the path analysis confirmed the results from correlational analyses and showed that verbal flexibility strongly predicted PF fluency, flexibility, and originality more than any other variable. Likelihood ratio test showed that using 1 or 3% cutoff for scoring originality did not significantly altered the results in both figural and verbal DT (vs. PF), while the likelihood ratio test showed significant differences between the figural and verbal DT. Finally, predictor variables in the verbal DT accounted for 40–58% of the variance in PF skills, whereas predictor variables in the figural DT accounted for 28–37% of the variance in PF skills. As suggested by experts in the field of PF, the role of flexibility in PF is a fertile area to be considered in future studies.

## Introduction

The significance of problem finding (PF) is widely recognized, and it is considered the first step in creative problem-solving efforts (e.g., Wallas, 1926; Osborn, 1953; Mumford et al., 1991; Treffinger and Isaksen, 2005). Some scholars have even considered PF more important than problem solving (e.g., Einstein and Infeld, 1938; Wertheimer, 1945; Mackworth, 1965; Csikszentmihalyi, 1988). A recent meta-analysis revealed a significant positive correlation between PF and creativity (Abdulla et al., 2020), of which the effect size is nearly the same as that reported in another meta-analysis (Kim, 2008) regarding the correlation between divergent thinking (DT) and creative achievement.

**FIGURE 1:**
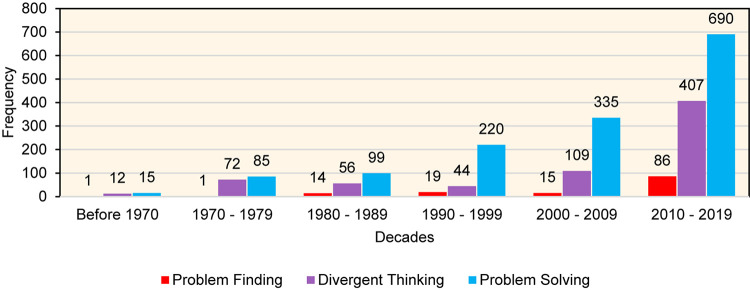
Number of publications focusing on problem finding, DT, and problem solving in the past five decades.

The history of studying PF began with the seminal work of Csikszentmihalyi and Getzels (1971), who aimed to assess PF in art students. Since then, the field of creativity research has displayed a growing interest in PF. However, this important cognitive process has not received as much attention from researchers in the field of creativity relative to DT or ideation. Figure 1 outlines the number of hits when the three terms (problem solving, DT, and PF) were searched from 1960 to 2020 in the following databases: ERIC, Academic Search Complete, Academic Search Premium, and ProQuest Central. Advanced searches were used, including the following combinations of keywords: (a) problem solving AND creativity OR creative, (b) DT AND creativity OR creative, and (c) all terminologies of PF reported in Abdulla and Cramond (2018; see page 202) AND creativity OR creative. The search only included titles, and redundant works were eliminated.

Although the results clearly indicate that research on PF is growing (e.g., Abdulla et al., 2020; Rubenstein et al., 2020a,b; AlSaleh et al., 2021), and that it is increasing at a higher rate relative to other research, the number of papers overall is still relatively small. This is considered a problem for several reasons. First, PF is an essential element in all well-known models of the creative process, such as Wallas’s four-stage model (Wallas, 1926), which includes (a) *preparation*, (b) incubation, (c) illumination, and (d) verification; Osborn-Parnes’s creative problem-solving model (Parnes, 1967), which includes (a) *fact finding*, (b) solution finding, and (c) acceptance finding; Basadur’s (1995) model which includes (a) PF, (b) problem solving, and (c) solution implementation, and Mumford et al.’s (1991) model of creative problem solving, which includes (a) *problem construction*, (b) information gathering, (c) concept selection, (d) conceptual combination, (e) idea generation, (f) idea evaluation, and (g) implementation planning. It should be noted that the role of PF in creative problem solving does not stop when the novel problem is identified. Rather, the problem solver might revisit and reformulate the problem after learning more about it (Runco, 1994b).

Second, some definitions of creativity explicitly include the concept of PF (e.g., Torrance, 1966; Cropley, 2011; Runco, 2014), and descriptions of how the concept is part of the creativity complex (Runco, 1994b). Third, advancing innovation and creativity for the benefit of business and society depends on people who can sense gaps in knowledge, discover ill-defined problems, and solve them in a novel way. Ma’s (2009) meta-analysis indicated that PF was the greatest predictor of creativity, which supports this last assertion. Additional support can be found in the work by Scott et al. (2004) which suggests that training of the PF process is the most effective in improving creativity.

However, this is not to say that other cognitive processes involved in creativity and creative thinking are less important than PF. The argument posited by this point is that PF is an essential cognitive ability that deserves more attention from researchers in the field of creativity.

After six decades of systematically studying PF, researchers have a deeper understanding of the relationship between PF and creativity as a whole and with specific cognitive processes, such as inter and intrapersonal evaluation (Mumford et al., 1991; Runco, 1994a,b; Basadur, 1995), problem solving (Arlin, 1975; Bouchard and Drauden, 1976; Carson and Runco, 1999), selective encoding and combination, selective comparison (Brugman, 1995), information search (Harms et al., 2020), and DT (e.g., Csikszentmihalyi and Getzels, 1971; Arlin, 1975; Artley et al., 1980; Wakefield, 1985; Chand and Runco, 1993; Hoover, 1994).

Of these cognitive processes, creativity literature suggests that DT is associated with PF–and many researchers have sought to explore this association (Arlin, 1975; Wakefield, 1985; Hoover and Feldhusen, 1990; Chand and Runco, 1993; Ambrosio, 1994; Hoover, 1994; Reiter-Palmon et al., 1997; Runco and Acar, 2010). However, these studies have yielded conflicting results (see Table 1), which could have been caused by (a) the DT and PF assessments that were used in the studies, (b) the DT skills that were targeted (i.e., fluency, flexibility, and originality), and (c) the nature of the task (verbal vs. figural tasks). The present study focuses on the latter two issues. Section “Literature Review: PF and DT” below illuminates the major findings from previous studies that have investigated the association between PF and DT. The section also demonstrates how the results differed according to the DT index that was studied and the nature of the tasks in the DT tests that were employed.

**TABLE 1:** Selected studies focusing on the relationship between DT and PF.

**Authors**	**DT Test**	***N***	**DT Skills**	**Task Nature**	**Main Findings**
Frederiksen and Evans (1974)	Consequences Test	395	Fluency	Verbal	The correlation between fluency and five formulating hypotheses (FH) tests ranged from 0.05 to 0.34.
Arlin (1975)	TTCT Puzzles Test	60	Expressional fluency Associational fluency Ideational fluency Adaptive flexibility Spontaneous flexibility Elaboration	Verbal Figural	A positive correlation was found between PF quality and (a) elaboration, (b) adaptive flexibility, and (c) expressional fluency; a negative correlation was found between PF quality and (a) spontaneous flexibility, (b) ideational fluency, and (c) associational fluency. A positive relationship was found between PF quantity and (a) spontaneous flexibility, (b) ideational fluency, and (c) associational fluency; PF quantity was negatively associated with (a) elaboration, (b) spontaneous flexibility, and (c) expressional fluency.
Artley et al. (1980)	TTCT	84	Composite	Verbal	TTCT positively correlated with PF quantity and negatively correlated with PF quality.
Wakefield (1985)	DT Test developed by the author	23	Composite	Figural	DT and PF were positively correlated.
Ambrosio (1994)	TTCT	60	Composite	Verbal	DT and PF quality were negatively correlated, while DT, PF, and PF quantity were positively correlated.
Chand and Runco (1993)	Presented Problems Test (PP) Problem Generation Test (PG) Discovered DT Test (DP)	80	Fluency Originality	Verbal	The PG test was positively correlated with fluency and originality for both PP and DP (ranging from *r* = 0.31 to 0.51).
Hoover (1994)	TTCT	40	Fluency Flexibility Originality	Verbal	TTCT fluency was positively correlated with fluency in the FH test. TTCT flexibility was positively correlated with fluency and originality in the FH test; no significant relationship was found between TTCT originality and FH fluency and originality.
Reiter-Palmon et al. (1997)	Consequences test (Guilford, 1966)	195	Ideational fluency Category Combination	Verbal	A negative correlation was found between PF and DT.
Lee and Cho (2007)	TTCT	115	Composite	Verbal	The ill-structured PF task was negatively correlated with DT, while PF and DT were positively correlated with the moderate problems.
Runco and Acar (2010)	Uses test	81	Fluency Originality	Verbal	DT fluency and originality were positively correlated with PF fluency and originality.
Jaarsveld et al. (2012)	Test for Creative Thinking–Drawing Production)	205	Composite	Figural	DT and PF were not significantly associated.

## Literature Review: PF and DT

After examining previous studies that investigated the association between PF and DT, this study noted that (a) most studies used a verbal DT when investigating the association, (b) fluency was the most studied DT index regarding the relationship between DT and PF (compared with flexibility, originality, and elaboration), (c) few studies examined the possible differences of the association in terms of nature of the DT task (i.e., verbal vs. figural), and (d) no studies explicitly aimed to understand which DT index is more strongly associated with PF.

The only study that considered both verbal and non-verbal DT tests in DT’s relationship with PF was Getzels and Smilansky (1983), who investigated the content and quality of problems that high school students formulated. Getzels and Smilansky’s (1983) main findings indicated that verbal originality (*r* = 0.23) and verbal flexibility (*r* = 0.18) were significantly associated with PF, and that no significant correlation was found with verbal fluency. Figural fluency, flexibility, and originality did not significantly correlate with PF, indicating that the association between DT and PF differs based on task nature.

Studies that employed verbal DT tests included Arlin (1975); Artley et al. (1980), Lee and Cho (2007), and Runco and Okuda (1988). Arlin’s (1975) study–which aimed to propose a cognitive process model of PF–reported a positive relationship between PF quality and adaptive flexibility (*r* = 0.26), and a negative relationship between PF quality and spontaneous flexibility (*r* = −0.09) and fluency (*r* = −0.18).

Artley et al. (1980) examined the association between verbal fluency, which was measured according to the Torrance Test of Creative Thinking (TTCT) and PF fluency. The results revealed a moderately significant correlation (*r* = 0.47) between the two variables. Artley et al. (1980) concluded that PF is independent from creativity, as assessed by DT tests. Runco and Okuda (1988) reached a similar conclusion after administering verbal DT and verbal PF tests: the correlation between the two variables was positively significant (*r* = 0.32). They concluded that “problem-finding skills is by definition statistically independent of problem-solving” (Runco and Okuda, 1988, p. 217). Runco and Okuda (1988) recognized the importance of considering other DT indices–more specifically, flexibility and originality–and they called for future research that examines the association between these other DT indices and PF. Finally, in a more recent study, Lee and Cho (2007) examined the association between PF using ill-structured problem situation tasks and TTCT. The verbal form of the TTCT was used, and the scholars reported a composite score for the verbal battery. A negative correlation (*r* = −0.17) was found between the DT and PF.

Some studies used a figural DT, such as those by Ambrosio (1994) and Wakefield (1985). Ambrosio’s (1994) study examined the PF differences between freshman and senior undergraduate students using the figural form of the TTCT. Ambrosio (1994) reported a negative relationship between DT and PF quality (*r* = −0.08) and a significant positive relationship between DT and PF quantity (*r* = 0.41). Ambrosio (1994) interpreted these results as demonstrating that DT is distinct from PF. Finally, Wakefield (1985) studied the association between figural DT (i.e., Wallack and Kogan’s pattern meaning and line meaning tests) and figural PF. Only fluency was scored for the DT and PF tasks. Wakefield (1985) reported a high correlation between DT and PF (*r* = 0.75) and concluded that “freedom to discover and solve problems appears to be the primary condition of creative performance” (p. 268). Moreover, Wakefield (1985) asserted that responses to presented problems (such as those used in DT tests) are marginally related to creativity, unlike PF tasks.

### PF and DT: Summary

As discussed above, and as shown in Table 1, only one study examined the relationship between PF and elaboration (i.e., Arlin, 1975), and only two studies examined the relationship between PF and flexibility (Arlin, 1975; Hoover, 1994). These study results suggest that the specific DT measure and index used (verbal or figural; fluency, flexibility, or originality) can affect the results (Hornberg and Reiter-Palmon, 2017; Reiter-Palmon et al., 2019). Further, much of the research that focuses on DT tasks tended to use fluency (Runco and Acar, 2012). Therefore, one of the present study’s contributions is the evaluation of flexibility and originality, in addition to fluency. Flexibility could be especially critical for PF. According to Mumford et al. (1994, p. 30), “Flexibility in applying selection and screening strategies may contribute to individual differences in PF skills.” In addition to evaluating the relationships with different DT indices, the present study also determined whether the relationship between DT and PF differs according to the nature of the DT task (verbal or figural). Finally, the present study also employed a more advanced path analysis method to understand the association between DT and PF. Based on the literature review presented in this section, the present study’s research hypotheses were determined as the following:

H1: The relationship between DT and PF differs according to the DT index (i.e., fluency, flexibility, and originality).
H2: The relationship between DT and PF will differs based on the nature of the DT task (figural or verbal).

## Materials and Methods

### Participants and Procedures

The participants included 90 students in the sixth grade (42 boys and 48 girls). We randomly collected data from two public schools after receiving the official approval from the Directorate for Scientific Research at the Ministry of Education. Prior to data collection, each participant was asked to read and sign a consent form prepared by the authors of this study.

Before the tests were administered, the first author visited the two schools and spent one class period (40 min) with the students to create familiarity with them (no DT or PF activities were discussed). By the end of this meeting, the author informed the study participants of a subsequent visit in the next week to administer some activities that could show the students’ creativity. The tests were administered in the second class/session (8:45 am), as recommended by the teachers. The participants were informed that they will obtain the results of their “creative activity score” after the authors had scored them. The tests were conducted in a game-like condition (untimed), following the results of a recent meta-analysis that showed that the untimed condition significantly improves students’ performance in DT tests (Said-Metwaly et al., 2020).

### Instruments

#### Uses Test

We administered three tasks from the Uses test (Wallach and Kogan, 1965): (a) uses for a spoon, (b) uses for a wheel, and (c) uses for a toothbrush. The directions for the task are as follows:

In this game, I am going to name an object–any kind of object, like a light bulb or the floor–and it will be your job to tell lots of different ways that the object could be used. Any object can be used in a lot of different ways. Remember, think of all the different ways you could use the object that I mentioned (Wallach and Kogan, 1965, p. 31).

Responses to the Uses test were scored for fluency, flexibility, and originality. A total of 199 *different* ideas were generated for the spoon task, 124 different ideas for the wheel task, and 181 different ideas for the toothbrush task. Fluency was defined as the number of different ideas related to a given stimulus. Flexibility was defined as the number of different categories or shifts between ideas. Based on participants’ responses, 12 categories were created for the spoon task, 11 for the wheel task, and eight for the toothbrush task (see Table 2). Finally, since some studies found that using different cutoff criteria can results in different findings, originality was scored based on both, 1% cutoff criterion and 3% cutoff criterion (Reiter-Palmon et al., 2019).

**TABLE 2:** Flexibility categories for verbal and figural tests.

**Task Nature**	**Categories**
***Verbal***	
Spoon	(1) Carry or move things, (2) care and beauty, (3) design and decoration, (4) artistic uses, (5) scientific uses, (6) recycling, (7) food and kitchen uses, (8) clean/cleaning, (9) stationery material, (10) game/use it in a game, (11) school/study uses, and (12) other uses.*
Wheel	(1) Carry or move things, (2) recycling and manufacturing, (3) decoration, (4) blocks, (5) shelter for pets, (6) container, (7) sport uses, (8) artistic uses, (9) musical instrument, (10) play and games, and (11) other uses.*
Toothbrush	(1) Cleaning, (2) care and beauty, (3) recycling, (4) artistic uses, (5) food and kitchen uses, (6) stationery material, (7) hanger, and (8) other uses.*
***Figural***	
Spiral	(1) Ideas/thoughts, (2) ropes, (3) nature (e.g., air, steam, and smoke), (4) path/road, (5) food, (6) letters/numbers, (7) games, (8) tools, (9) trees, and (10) others.*
Blocks	(1) Places, (2) games, (3) geometric figures, (4) mathematics, (5) roads/crossroads, (6) machines, (7) part of the human body, (8) tools, (9) phone/tablets, (10) letter or words, and (11) others.*
Lines	(1) Road, (2) sorting things, (3) architecture, (4) stick/cane, (5) artistic and design uses, (6) furniture, (8) games, (9) musical instruments, (10) levels, and (11) others.*

*Others = all responses that did not fit any of the mentioned categories.

#### Figural Test

We administered the Figural test from the Runco Creativity Assessment Battery (2020), which consists of three tasks, similar to the Wallach and Kogan Line Meaning test (Wallach and Kogan, 1965, p. 36). The Figural test includes three shapes: Spiral, Blocks, and Lines. The directions are as follows:

Look at the figure below. What do you see? List as many things as you can that this figure might be or represent. This is NOT a test. Think of this as a game and have fun with it! The more ideas you list, the better (Runco et al., 2016, p. 6).

The Figural test was also scored for fluency, flexibility, and originality. A total of 105 different ideas were generated for the first task (Spiral), 111 for the second task (Blocks), and 53 for the third task (Lines). For flexibility, 10 categories were created in the Spiral task, 11 categories in the Blocks task, and 11 categories in the Lines task. Originality was scored using the same method as the Uses test (i.e., 1 and 3% cutoffs).

#### Problem Generation Test

We used the Problem Generation (PG) test to assess participants’ PF ability (Okuda et al., 1991)^1^. The PG test consists of three open-ended tasks that require participants to list as many problems as they can. Participants were asked to generate as many problems as they can (real or hypothetical) without any instruction to evaluate any of their ideas, or that their ideas will be evaluated. The three PG tasks are related to (a) home and school, (b) life situations, and (c) health and well-being. An example of a PG task is as follows:

List problems along with your friends, peers, or schoolmates (any individual who is approximately the same age as your-self). These problems can be real, or they can be hypothetical and imaginary. Do not limit yourself; the more problems you can list, the better. You can think of problems that exist now or those that might exist in future.

The PG test was scored for fluency, flexibility, and originality. The participants generated 235, 253, and 269 different ideas in the first, second, and third tasks, respectively. For flexibility, 11 categories were created in the first task (home and school), 12 categories in the second task (life situation), and 13 categories in the third task (health and well-being). The same method was utilized for scoring originality in the PG test (i.e., 1 and 3% cutoffs).

Finally, the students were asked to fill out a demographic questionnaire with items on their age and sex.

## Results

### Reliability

Both the interclass correlation (ICC) and internal consistency reliability were calculated for the three tests that were used in the study. As mentioned earlier, DT verbal and figural tests (as well as PG tests) were scored for fluency, flexibility, and originality. Table 3 lists the reliability coefficients for the three tests. For the ICC, a two-way mixed-model absolute agreement was calculated.

**TABLE 3:** Interclass and internal consistency reliability coefficients of study instruments.

	**ICC**	**Internal Consistency**
Fluency–Uses	0.81	0.93
Flexibility–Uses	0.84	0.85
Originality–Uses (3%)	0.83	0.84
Originality–Uses (1%)	0.81	0.89
Fluency–Figural	0.53 (0.71)*	0.61 (0.73)*
Flexibility–Figural	0.50 (0.68)*	0.64 (0.69)*
Originality–Figural (3%)	0.51 (0.62)*	0.55 (0.62)*
Originality–Figural (1%)	0.59 (0.76)*	0.45 (59)*
Problem Generation–Fluency	0.87	0.88
Problem Generation–Fluency	0.80	0.71
Problem Generation–Originality (3%)	0.87	0.87
Problem Generation–Originality (3%)	0.88	0.83

*After removing the Lines task.

*ICC, Interclass Correlation.*

As moderate reliability coefficients were observed in the figural test, this study examined each task of the figural test. The *lines* task demonstrated lower reliability coefficients in the figural test. After removing the lines test, the reliability coefficients in the figural test improved. Therefore, the lines task scores were eliminated in all subsequent analyses.

#### Correlation Analysis

As outlined in Table 4, the pattern of correlations between DT and PF differed in the verbal and figural tests. In the verbal DT, the highest correlation was found between verbal flexibility and PF (*r* = 0.71–0.76, *p* < 0.001), followed by verbal fluency and PF (*r* = 0.68–0.71, *p* < 0.001) and verbal originality and PF (*r* = 0.55–0.61, *p* < 0.001). This pattern remained the same regardless of whether the 1% cutoff or 3% cutoff was used as the originality criterion.

**TABLE 4:** Correlations between study variables (*n* = 90).

	**Fluency**	**Flexibility**	**Originality**	**Fluency**	**Flexibility**	**Originality**	**Fluency**	**Flexibility**	**Originality**
	**(Uses)**	**(Uses)**	**(Uses)**	**(Figures)**	**(Figures)**	**(Figures)**	**(PG)**	**(PG)**	**(PG)**
Fluency (Uses)		0.88**	0.93**	0.16	0.18	0.26*	0.70**	0.69**	0.71**
Flexibility (Uses)	0.88**		0.74**	0.12	0.14	0.25*	0.74**	0.76**	0.72**
Originality (Uses)	0.90**	0.70**		0.17	0.19	0.29**	0.66**	0.61*	0.68**
Fluency (Figures)	0.16	0.12	0.15		0.91**	0.70**	0.14	0.18	0.16
Flexibility (Figures)	0.18	0.14	0.17	0.91**		0.63**	0.15	0.20	0.17
Originality (Figures)	0.24*	0.23*	0.23*	0.63**	0.57*		0.26*	0.30**	0.28**
Fluency (PG)	0.70**	0.74**	0.61**	0.14	0.15	0.27*		0.93**	0.98**
Flexibility (PG)	0.69**	0.76**	0.55**	0.18	0.20	0.31**	0.93**		0.91**
Originality (PG)	0.68**	0.71**	0.60**	0.15	0.15	0.30**	0.96**	0.90**	

*The results for the 1% originality cutoff are displayed above the diagonal, and the results for the 3% cutoff are displayed below the diagonal.*

Uses = uses test; PG = problem generation test.

*p < 0.05; **p < 0.01.

Further, the magnitude of the correlation between figural DT and PF was much lower than that of verbal DT. More specifically, the highest correlation was found between figural originality and PF (*r* = 0.27–0.31, *p* < 0.05), followed by figural flexibility and PF (*r* = 0.15–0.20) and figural fluency and PF (*r* = 0.14–0.18). Again, the different cutoff criteria for originality did not alter the results.

#### Path Analysis

**FIGURE 2:**
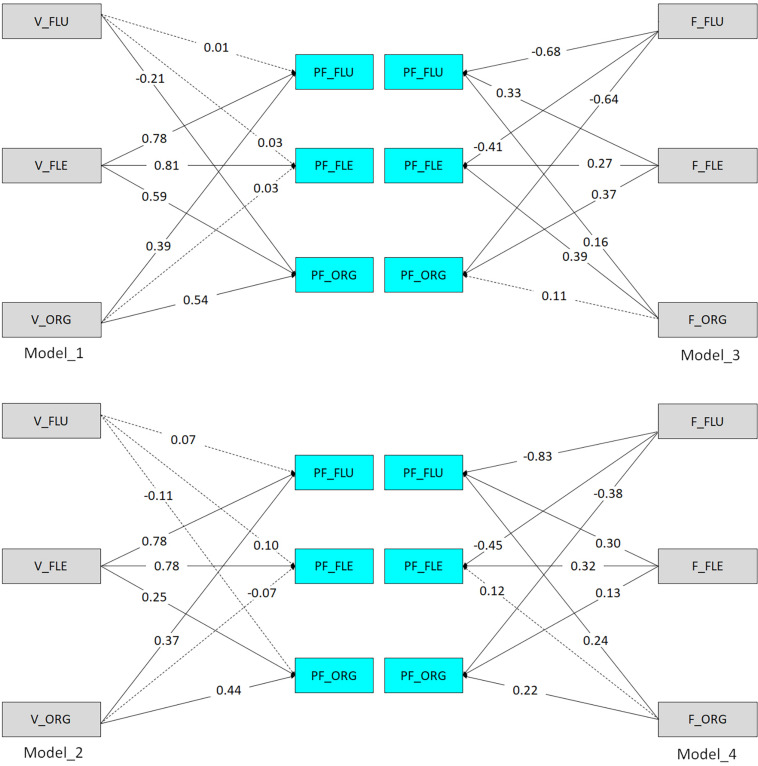
Path analysis models for the study variables. V, verbal; F, figural; PF, problem finding; FLU, fluency; FLE, flexibility; ORG, originality.

While correlational analysis allows for a better understanding of the bivariate relationship between DT and PF, examining all the relationships at the same time is important as well. After creating the models and selecting the required path directions, the maximum likelihood method was used to estimate the parameters of the structural equation model. The present study then analyzed how verbal and figural DT (fluency, flexibility, and originality at the 3% cutoff) predicted PF (fluency, flexibility, and originality at the 3% cutoff), and how verbal and figural DT (fluency, flexibility, and originality at the 1% cutoff) predicted PF (fluency, flexibility, and originality at the 1%). The four path analysis models are illustrated in Figure 2.

As displayed in Figure 2, the standardized path coefficients (β) and *t* values were observed to be between the DT indices and PF. These values indicate that the model adequately fits the data. After examining the fit indices χ^2^/*df*, RMSEA, GFI, AGFI, and NFI, the values indicated that the models adequately fits the data. Table 5 showed the four models.

**TABLE 5:** Goodness-of-fit indices for the different models.

***Model***	**χ^2^**	***df***	**χ^2^/*df***	***RMSEA 90% CI***	***GFI***	***AGFI***	***NFI***
*Model_1 (DT Verbal and Originality 3%)*	18.40	16	1.15	0.047 [0.044, 0.049]	0.96	0.95	0.96
*Model_2 (DT Verbal and Originality 1%)*	20.59	18	1.14	0.050 [0.048, 0.053]	0.94	0.92	0.93
*Model_3 (DT Figural and Originality 3%)*	50.95	18	2.83	0.072 [0.069, 0.075]	0.94	0.91	0.90
*Model_4 (DT Figural and Originality 1%)*	56.17	20	2.81	0.083 [0.080, 0.086]	0.92	0.89	0.88

*RMSEA, root mean square error of approximation; CI, confidence interval; GFI, Goodness of Fit Index; AGFI, Adjusted Goodness of Fit Index; NFI, Normed Fit Index.*

First, Model 1 indicated that the predictor variables accounted for 56, 58, and 43% of the variance in PF fluency, flexibility, and originality, respectively. The results verified the model and confirmed that verbal flexibility strongly affected: (a) PF flexibility (β = 0.81, *p* < 0.001), (b) PF fluency (β = 0.78, *p* < 0.001), and (3) PF originality (β = 0.59, *p* < 0.001). Finally, verbal originality showed significant effects on: (a) PF originality (β = 0.54, *p* < 0.001) and (b) PF fluency (β = 0.39, *p* < 0.001).

Second, Model 2 showed that the predictor variables accounted for 56, 40, and 53% of the variance in PF fluency, flexibility, and originality, respectively. The results verified the model and confirmed that verbal flexibility strongly affected: (a) PF flexibility (β = 0.78, *p* < 0.001), (b) PF fluency (β = 0.78, *p* < 0.001), and (3) PF originality (β = 0.25, *p* < 0.001). Finally, verbal originality has a significant effect on: (a) PF originality (β = 0.44, *p* < 0.001) and (b) PF fluency (β = 0.37, *p* < 0.001).

Third, Model 3 showed that the predictor variables accounted for 35%, 37%, and 35% of the variance in PF fluency, flexibility, and originality, respectively. The results verified the model and confirmed that figural flexibility strongly affected: (a) PF originality (β = 0.37, *p* < 0.001), (b) PF fluency (β = 0.33, *p* < 0.001), and (3) PF flexibility (β = 0.27, *p* < 0.001). Figural originality has a significant effect on: (a) PF flexibility (β = 0.39, *p* < 0.001), and (b) PF fluency (β = 0.16, *p* < 0.05).

Finally, Model 4 indicated that the predictor variables accounted for 33%, 30%, and 28% of the variance in PF fluency, flexibility, and originality, respectively. The results verified the model and confirmed that figural flexibility strongly affected: (a) PF flexibility (β = 0.32, *p* < 0.001), (b) PF fluency (β = 0.30, *p* < 0.001), and (3) PF originality (β = 0.13, *p* < 0.05). Finally, figural originality has a significant effect on: (a) PF flexibility (β = 0.24, *p* < 0.001), and (b) PF originality (β = 0.22, *p* < 0.001; see Table 6).

**TABLE 6:** Path analysis of DT indices with PF.

**Variable**	***Model_1 (Originality 3%)***									***Model_2 (Originality 1%)***								
	**Fluency (PF)**			**Flexibility (PF)**			**Originality (PF)**			**Fluency (PF)**			**Flexibility (PF)**			**Originality (PF)**		
	**β**	***SE***	***t***	***B***	***SE***	***t***	**β**	***SE***	***t***	***B***	***SE***	***t***	**β**	***SE***	***t***	**β**	***SE***	***t***
***DT Verbal***																		
Fluency	0.01	0.068	0.15	0.03	0.065	0.46	−0.21	0.065	−3.23**	0.07	0.067	1.04	0.10	0.064	1.56	−0.11	0.065	−1.69
Flexibility	0.78	0.066	11.82**	0.81	0.064	12.66**	0.59	0.066	8.94**	0.78	0.064	12.19**	0.78	0.063	12.38**	0.25	0.063	3.97**
Originality	0.39	0.069	5.65**	0.03	0.064	0.47	0.54	0.067	8.06**	0.37	0.066	5.61**	−0.07	0.067	−1.04	0.44	0.063	6.98**
*R*^2^	0.56			0.58			0.43			0.56			0.40			0.53		

**Likelihood ratio test between Model_1 and Model_2 Δχ^2^ = 2.19, Δ*df* = 2, *p* = 0.335**																		
	***Model_3 (Originality 3%)***									***Model_4 (Originality 1%)***								
	**Fluency (PF)**			**Flexibility (PF)**			**Originality (PF)**			**Fluency (PF)**			**Flexibility (PF)**			**Originality (PF)**		
***DT Figural***																		
Fluency	−0.68	0.069	−9.86**	−0.41	0.067	−6.12*	−0.64	0.069	9.28**	−0.83	0.067	−12.39**	−0.45	0.067	−6.72**	−0.38	0.067	5.67**
Flexibility	0.33	0.069	4.78**	0.27	0.069	3.91**	0.37	0.067	5.52**	0.30	0.068	4.41**	0.32	0.068	4.71**	0.13	0.065	2.00*
Originality	0.16	0.067	2.38*	0.39	0.066	5.91**	0.11	0.068	1.62	0.24	0.068	3.53**	0.12	0.069	1.74	0.22	0.068	3.24**
*R*^2^	0.35			0.37			0.35			0.33			0.30			0.28		
**Likelihood ratio test between Model_3 and Model_4 Δχ^2^ = 1.10, Δ*df* = 2, *p* = 0.073**																		
Likelihood ratio test between Model_1 and Model_3 Δχ^2^ = 2.77, Δ*df* = 2, *p* = 0.008										Likelihood ratio test between Model_2 and Model_4 Δχ^2^ = 2.73, Δ*df* = 2, *p* = 0.001								

β = standardized path coefficients; SE = standard error; DT = divergent thinking; PF = problem finding. *p < 0.05; **p < 0.01.

Likelihood ratio test showed that there were no significant differences between Models 1 and 2, (Δχ^2^ = 2.19, Δ*df* = 2, *p* = 0.335). Furthermore, there were no significant differences between Models 3 and 4, (Δχ^2^ = 1.10, Δ*df* = 2, *p* = 0.073), indicating that using different cutoff scores for originality (1 vs. 3%) did not affect the results. As for the differences between verbal and figural DT, the results indicated that there were significant differences between Models 1 and 3 (DT Verbal vs. DT Figural; 3% *cutoff originality*) in favor of Verbal DT (Δχ^2^ = 2.77, Δ*df* = 2, *p* = 0.008). Moreover, there were significant differences between Models 2 and 4 (DT Verbal vs. DT Figural; 1% *cutoff originality*) in favor of Verbal DT (Δχ^2^ = 2.73, Δ*df* = 2, *p* = 0.001). It can thus be concluded that the nature of the task significantly influenced the relationship between PF and DT, such that verbal tasks were more strongly related to PF.

## Discussion

Studies on PF have suggested that some cognitive processing skills are related to PF, and have also paid attention to DT along with PF. Previous research has examined the association between PF and DT, however, none of these studies have explicitly aimed to examine multiple DT indices and tasks and the relative association with PF. The results of our study showed that flexibility, which was neglected in previous works on PF and DT, was highly correlated with PF compared with fluency and originality, especially when verbal tasks were administered. In the path models, flexibility of verbal DT was the best predictor of the PF task performance. Arlin (1975) described problem finders as “flexible thinkers.” Our findings support this claim and suggest that good problem finders are characterized by an ability to shift between ideas and keep formulating different ideas to find a novel problem that needs to be solved. However, more studies are needed to elucidate fully the role of flexibility in PF.

The other important finding in the present study is the difference in the relation between PF and DT based on the task nature. We found that PF can be better assessed using verbal tasks than figural ones. This pattern of results included both the correlational analysis and the path models. This finding is partly supported by Abdulla et al. (2020), in which PF in the writing domains is highly correlated with creativity (*r* = 0.36) compared with art (*r* = 0.20). One issue that future studies might consider is whether highly capable problem finders are overrepresented in writing vs. in other domains. It is possible that the strong correlations between PF and DT in the writing domain is in part a result of both requiring writing as the mode of generated responses. Previous research suggests that the task and index of measurement is important in understanding these types of relationships (Reiter-Palmon et al., 2019; Reiter-Palmon and Schoenbeck, 2020). Additional research evaluating whether these relationships hold with other verbal DT measures such as consequences as well as other figural DT measures would help clarify this issue.

The path model also suggests that originality of verbal DT responses was important for predicting PF. Specifically, originality was predictive of PF fluency and PF originality. Interestingly, the results from the path analysis suggest that verbal DT fluency was either minimally predictive or non-significant. When it was predictive, the relationship that was obtained was negative, which suggests that when other dimensions of DT scoring are included, fluency is less important or even may have a negative effect. This further indicates that it is not sufficient to evaluate DT fluency when trying to understand the relationship between DT and other creativity measures and processes, such as PF.

## Limitations

It is important to note that PF is not a single process. Many scholars have differentiated known PF skills, such as problem definition, problem identification, problem construction, and problem discovery (e.g., Getzels, 1982; Mumford et al., 1994; Runco, 1994b). The differences between these and other PF-related skills lie in (a) how well- or ill-defined a problem is and (b) the degree to which ideation and evaluation are required (Abdulla and Cramond, 2018). In our study, PF was operationalized as *problem discovery*, which has been considered as the highest level of PF hierarchy (Abdulla and Cramond, 2018). We asked the participants to produce as many ideas as they could think of for *different* and *novel* problems related to home and school, life situations, and health and well-being. They were explicitly instructed to think of real *or* hypothetical problems, and to think of problems that exist *or* those that might exist in the future. Previous work on instructions indicates that the focus of instructions can have a significant impact on the outcomes associated with DT measures (Runco et al., 2005; Nusbaum et al., 2014; Acar et al., 2020). The focus of the instructions is on both different and novel ideas may be one reason why flexibility was such an effective predictor. This point is crucial because different results might be attributed to the level of ideation and evaluation required in the PF task (Lee and Cho, 2007). Thus, our findings might not be generalized to all PF tasks, except those that require problem discovery where there is limited role for evaluation. The nature of the specific PF task, focusing on generation, may also indicate some degree of common method bias. This would be particularly the case if fluency was the only metric that was used for the study. However, since flexibility and originality were evaluated for both the DT and PF task, this is less of a concern. This study adds to our understanding of the relationship between PF and DT. Future research should address some of the limitations noted here such as including additional PF tasks. In addition, evaluating the effects of task instructions for both PF and DT tasks by varying task instructions would be beneficial. Finally, additional research should evaluate the relationships in other populations such as adolescents and adults.

## Data Availability Statement

The raw data supporting the conclusions of this article will be made available by the authors, without undue reservation.

## Ethics Statement

The studies involving human participants were reviewed and approved by Directorate for Scientific Research at the Ministry of Education, Bahrain. Written informed consent to participate in this study was provided by the participants’ legal guardian/next of kin.

## Author Contributions

AMA originated the research idea and wrote the manuscript with support from RR-P. AMA wrote the introduction and the results. RR-P wrote the discussion and reviewed the introduction. ZS collected the data and scored the assessments (two divergent thinking tests and a problem construction test). AMA and ZS calculated in interclass correlation reliability. AAA wrote the method section and analyzed the data. All authors contributed to the article and approved the submitted version.

## Conflict of Interest

The authors declare that the research was conducted in the absence of any commercial or financial relationships that could be construed as a potential conflict of interest.

## Footnotes

1 http://creativitytestingservice.com
